# Methyl 3-benzyl­idenecarbazate

**DOI:** 10.1107/S1600536809045012

**Published:** 2009-10-31

**Authors:** Yu-Feng Li, Hai-Xing Liu, Fang-Fang Jian

**Affiliations:** a Microscale Science Institute, Department of Chemistry and Chemical Engineering, Weifang University, Weifang 261061, People’s Republic of China; b Microscale Science Institute, Weifang University, Weifang 261061, People’s Republic of China

## Abstract

In the crystal of the title compound, C_9_H_10_N_2_O_2_, the mol­ecules are linked by N—H⋯O hydrogen bonds, generating S(4) chains propagating in [010].

## Related literature

For background to Schiff bases, see: Cimerman *et al.* (1997 ▶).
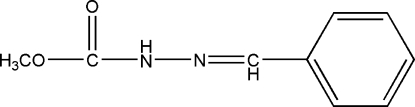


## Experimental

### 

#### Crystal data

C_9_H_10_N_2_O_2_
*M* *_r_* = 178.19
Orthorhombic, 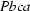

*a* = 12.278 (3) Å
*b* = 7.8035 (16) Å
*c* = 19.466 (4) Å
*V* = 1865.1 (7) Å^3^
*Z* = 8
Mo *K*α radiation
μ = 0.09 mm^−1^
*T* = 293 K
0.25 × 0.21 × 0.19 mm

#### Data collection

Bruker SMART CCD area-detector diffractometer
Absorption correction: none
16349 measured reflections
2132 independent reflections
1693 reflections with *I* > 2σ(*I*)
*R*_int_ = 0.048

#### Refinement

*R*[*F* ^2^ > 2σ(*F* ^2^)] = 0.040
*wR*(*F* ^2^) = 0.147
*S* = 1.08
2132 reflections
119 parameters
H-atom parameters constrained
Δρ_max_ = 0.23 e Å^−3^
Δρ_min_ = −0.18 e Å^−3^

### 

Data collection: *SMART* (Bruker, 1997 ▶); cell refinement: *SAINT* (Bruker, 1997 ▶); data reduction: *SAINT*; program(s) used to solve structure: *SHELXS97* (Sheldrick, 2008 ▶); program(s) used to refine structure: *SHELXL97* (Sheldrick, 2008 ▶); molecular graphics: *SHELXTL* (Sheldrick, 2008 ▶); software used to prepare material for publication: *SHELXTL*.

## Supplementary Material

Crystal structure: contains datablocks global, I. DOI: 10.1107/S1600536809045012/hb5195sup1.cif

Structure factors: contains datablocks I. DOI: 10.1107/S1600536809045012/hb5195Isup2.hkl

Additional supplementary materials:  crystallographic information; 3D view; checkCIF report

## Figures and Tables

**Table 1:** Hydrogen-bond geometry (Å, °)

*D*—H⋯*A*	*D*—H	H⋯*A*	*D*⋯*A*	*D*—H⋯*A*
N1—H1*A*⋯O2^i^	0.86	2.04	2.8986 (15)	179

Symmetry code: (i) 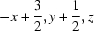.

**Fig. 1.**
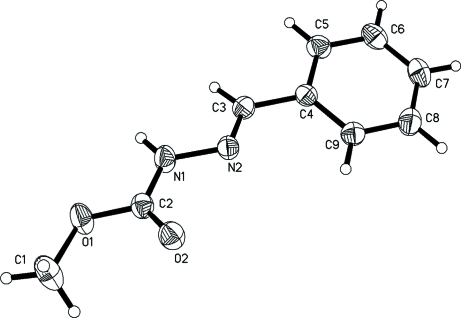
The structure of the title compound showing 30% probability displacement ellipsoids.

## supplementary crystallographic information

### Experimental

A mixture of benzaldehyde (0.1 mol), and methyl carbazate (0.1 mol) was stirred in refluxing ethanol (20 mL) for 4 h to afford the title compound (0.089 mol, yield 89%).  Colourless blocks of (I) were obtained by recrystallization from ethanol at room temperature.

### Refinement

H atoms were fixed geometrically and allowed to ride on their attached atoms, with C—H = 0.97 Å, and with *U*_iso_ = 1.2–1.5*U*_eq_.

### Crystal data

**Table:**

C_9_H_10_N_2_O_2_	*F*(000) = 752
*M**_r_* = 178.19	*D*_x_ = 1.269 Mg m^−^^3^
Orthorhombic, *P**b**c**a*	Mo *K*α radiation, λ = 0.71073 Å
Hall symbol: -P 2ac 2ab	Cell parameters from 1984 reflections
*a* = 12.278 (3) Å	θ = 3.5–27.5°
*b* = 7.8035 (16) Å	µ = 0.09 mm^−^^1^
*c* = 19.466 (4) Å	*T* = 293 K
*V* = 1865.1 (7) Å^3^	Block, colourless
*Z* = 8	0.25 × 0.21 × 0.19 mm

### Data collection

**Table:**

Bruker SMART CCD area-detector diffractometer	1693 reflections with *I* > 2σ(*I*)
Radiation source: fine-focus sealed tube	*R*_int_ = 0.048
graphite	θ_max_ = 27.5°, θ_min_ = 3.3°
φ and ω scans	*h* = −15→15
16349 measured reflections	*k* = −10→8
2132 independent reflections	*l* = −25→25

### Refinement

**Table:**

Refinement on *F*^2^	Secondary atom site location: difference Fourier map
Least-squares matrix: full	Hydrogen site location: inferred from neighbouring sites
*R*[*F*^2^ > 2σ(*F*^2^)] = 0.040	H-atom parameters constrained
*wR*(*F*^2^) = 0.147	*w* = 1/[σ^2^(*F*_o_^2^) + (0.1*P*)^2^] where *P* = (*F*_o_^2^ + 2*F*_c_^2^)/3
*S* = 1.08	(Δ/σ)_max_ = 0.001
2132 reflections	Δρ_max_ = 0.23 e Å^−^^3^
119 parameters	Δρ_min_ = −0.18 e Å^−^^3^
0 restraints	Extinction correction: *SHELXL97* (Sheldrick, 2008), Fc^*^=kFc[1+0.001xFc^2^λ^3^/sin(2θ)]^-1/4^
Primary atom site location: structure-invariant direct methods	Extinction coefficient: 0.011 (3)

### Special details

**Table:**

Geometry. All e.s.d.'s (except the e.s.d. in the dihedral angle between two l.s. planes) are estimated using the full covariance matrix. The cell e.s.d.'s are taken into account individually in the estimation of e.s.d.'s in distances, angles and torsion angles; correlations between e.s.d.'s in cell parameters are only used when they are defined by crystal symmetry. An approximate (isotropic) treatment of cell e.s.d.'s is used for estimating e.s.d.'s involving l.s. planes.
Refinement. Refinement of *F*^2^ against ALL reflections. The weighted *R*-factor *wR* and goodness of fit *S* are based on *F*^2^, conventional *R*-factors *R* are based on *F*, with *F* set to zero for negative *F*^2^. The threshold expression of *F*^2^ > σ(*F*^2^) is used only for calculating *R*-factors(gt) *etc*. and is not relevant to the choice of reflections for refinement. *R*-factors based on *F*^2^ are statistically about twice as large as those based on *F*, and *R*- factors based on ALL data will be even larger.

### Fractional atomic coordinates and isotropic or equivalent isotropic displacement parameters (Å^2^)

**Table:**

	*x*	*y*	*z*	*U*_iso_*/*U*_eq_	
O2	0.87392 (7)	0.13497 (11)	0.52542 (4)	0.0522 (3)	
N2	0.72993 (8)	0.24691 (12)	0.62265 (4)	0.0434 (3)	
O1	0.85158 (9)	0.38853 (12)	0.47171 (5)	0.0613 (3)	
C3	0.65739 (9)	0.31376 (15)	0.66062 (6)	0.0443 (3)	
H3A	0.6184	0.4077	0.6445	0.053*	
N1	0.74972 (9)	0.33172 (13)	0.56166 (5)	0.0505 (3)	
H1A	0.7125	0.4207	0.5504	0.061*	
C4	0.63373 (10)	0.24621 (14)	0.72925 (5)	0.0418 (3)	
C2	0.82860 (10)	0.27174 (15)	0.52044 (5)	0.0434 (3)	
C9	0.70485 (10)	0.13562 (16)	0.76285 (6)	0.0508 (3)	
H9A	0.7678	0.0986	0.7408	0.061*	
C7	0.58904 (13)	0.13493 (19)	0.86228 (7)	0.0599 (4)	
H7A	0.5747	0.0988	0.9069	0.072*	
C5	0.54021 (11)	0.29976 (18)	0.76341 (6)	0.0532 (3)	
H5A	0.4922	0.3747	0.7419	0.064*	
C8	0.68246 (13)	0.08046 (19)	0.82877 (6)	0.0596 (4)	
H8A	0.7304	0.0063	0.8508	0.072*	
C6	0.51809 (12)	0.24207 (18)	0.82934 (7)	0.0607 (4)	
H6A	0.4545	0.2765	0.8513	0.073*	
C1	0.93760 (15)	0.3429 (2)	0.42527 (8)	0.0764 (5)	
H1B	0.9484	0.4338	0.3928	0.115*	
H1C	0.9184	0.2397	0.4013	0.115*	
H1D	1.0036	0.3246	0.4507	0.115*	

### Atomic displacement parameters (Å^2^)

**Table:**

	*U*^11^	*U*^22^	*U*^33^	*U*^12^	*U*^13^	*U*^23^
O2	0.0572 (6)	0.0453 (5)	0.0541 (5)	0.0041 (4)	0.0082 (4)	0.0034 (3)
N2	0.0465 (6)	0.0427 (6)	0.0410 (5)	0.0010 (4)	0.0018 (4)	0.0047 (4)
O1	0.0771 (7)	0.0523 (6)	0.0546 (5)	0.0022 (5)	0.0182 (4)	0.0139 (4)
C3	0.0451 (6)	0.0414 (6)	0.0464 (6)	0.0032 (5)	−0.0009 (4)	0.0013 (5)
N1	0.0609 (6)	0.0439 (6)	0.0467 (6)	0.0103 (4)	0.0078 (4)	0.0111 (4)
C4	0.0426 (6)	0.0396 (6)	0.0431 (6)	−0.0032 (4)	0.0012 (4)	−0.0018 (5)
C2	0.0485 (7)	0.0411 (6)	0.0406 (6)	−0.0053 (5)	−0.0008 (4)	0.0020 (4)
C9	0.0478 (7)	0.0529 (7)	0.0517 (7)	0.0042 (5)	0.0020 (5)	0.0018 (5)
C7	0.0777 (9)	0.0568 (8)	0.0452 (7)	−0.0139 (7)	0.0074 (6)	−0.0002 (5)
C5	0.0488 (7)	0.0525 (7)	0.0584 (7)	0.0036 (5)	0.0066 (5)	0.0004 (5)
C8	0.0678 (9)	0.0585 (8)	0.0526 (7)	−0.0012 (6)	−0.0053 (6)	0.0085 (5)
C6	0.0614 (8)	0.0595 (8)	0.0613 (8)	−0.0038 (6)	0.0204 (6)	−0.0057 (6)
C1	0.0882 (11)	0.0756 (10)	0.0652 (9)	−0.0041 (8)	0.0305 (8)	0.0085 (8)

### Geometric parameters (Å, °)

**Table:**

O2—C2	1.2075 (15)	C9—H9A	0.9300
N2—C3	1.2694 (15)	C7—C6	1.367 (2)
N2—N1	1.3808 (13)	C7—C8	1.386 (2)
O1—C2	1.3453 (14)	C7—H7A	0.9300
O1—C1	1.4351 (18)	C5—C6	1.3868 (17)
C3—C4	1.4653 (16)	C5—H5A	0.9300
C3—H3A	0.9300	C8—H8A	0.9300
N1—C2	1.3420 (16)	C6—H6A	0.9300
N1—H1A	0.8600	C1—H1B	0.9600
C4—C9	1.3911 (18)	C1—H1C	0.9600
C4—C5	1.3912 (17)	C1—H1D	0.9600
C9—C8	1.3811 (18)		
			
C3—N2—N1	115.28 (10)	C6—C7—H7A	120.2
C2—O1—C1	115.48 (11)	C8—C7—H7A	120.2
N2—C3—C4	121.47 (11)	C6—C5—C4	120.43 (13)
N2—C3—H3A	119.3	C6—C5—H5A	119.8
C4—C3—H3A	119.3	C4—C5—H5A	119.8
C2—N1—N2	118.29 (10)	C9—C8—C7	120.42 (13)
C2—N1—H1A	120.9	C9—C8—H8A	119.8
N2—N1—H1A	120.9	C7—C8—H8A	119.8
C9—C4—C5	118.66 (11)	C7—C6—C5	120.52 (13)
C9—C4—C3	121.84 (11)	C7—C6—H6A	119.7
C5—C4—C3	119.43 (11)	C5—C6—H6A	119.7
O2—C2—N1	126.35 (10)	O1—C1—H1B	109.5
O2—C2—O1	123.97 (11)	O1—C1—H1C	109.5
N1—C2—O1	109.67 (10)	H1B—C1—H1C	109.5
C8—C9—C4	120.35 (12)	O1—C1—H1D	109.5
C8—C9—H9A	119.8	H1B—C1—H1D	109.5
C4—C9—H9A	119.8	H1C—C1—H1D	109.5
C6—C7—C8	119.60 (12)		

### Hydrogen-bond geometry (Å, °)

**Table:**

*D*—H···*A*	*D*—H	H···*A*	*D*···*A*	*D*—H···*A*
N1—H1A···O2^i^	0.86	2.04	2.8986 (15)	179

Symmetry codes: (i) −*x*+3/2, *y*+1/2, *z*.

## References

[bb1] Bruker (1997). *SMART* and *SAINT* Bruker AXS Inc., Madison, Wisconsin, USA.

[bb2] Cimerman, Z., Galic, N. & Bosner, B. (1997). *Anal. Chim. Acta*, **343**, 145–153.

[bb3] Sheldrick, G. M. (2008). *Acta Cryst.* A**64**, 112–122. 10.1107/S0108767307043930 18156677

